# Reformulation as an Integrated Approach of Four Disciplines: A Qualitative Study with Food Companies

**DOI:** 10.3390/foods7040064

**Published:** 2018-04-20

**Authors:** Annelies van Gunst, Annet J. C. Roodenburg, Ingrid H. M. Steenhuis

**Affiliations:** 1 Department of Food. HAS University of Applied Sciences, Onderwijsboulevard 221, 5223 DE ’s-Hertogenbosch, The Netherlands; a.roodenburg@has.nl; 2 Department of Health Sciences, Faculty of Science, VU University Amsterdam, Amsterdam Public Health Research Institute, De Boelelaan 1085, 1081 HV Amsterdam, The Netherlands; ingrid.steenhuis@vu.nl

**Keywords:** reformulation, food companies, food technology, nutrition and health, legislation, consumer perspectives

## Abstract

In 2014, the Dutch government agreed with the food sector to lower salt, sugar, saturated fat and energy in foods. To reformulate, an integrated approach of four disciplines (Nutrition & Health, Food Technology, Legislation, and Consumer Perspectives) is important for food companies (Framework for Reformulation). The objective of this study was to determine whether this framework accurately reflects reformulation processes in food companies. Seventeen Dutch food companies in the bakery, meat and convenience sector were interviewed with a semi-structured topic list. Interviews were transcribed, coded and analysed. Interviews illustrated that there were opportunities to lower salt, sugar and saturated fat (Nutrition & Health). However, there were barriers to replacing the functionality of these ingredients (Food Technology). Most companies would like the government to push reformulation more (Legislation). Traditional meat products and luxury sweet bakery products were considered less suitable for reformulation (Consumer Perspectives). In addition, the reduction of E-numbers was considered important. The important role of the retailer is stressed by the respondents. In conclusion, all four disciplines are important in the reformulation processes in food companies. Reformulation does not only mean the reduction of salt, saturated fat and sugar for companies, but also the reduction of E-numbers.

## 1. Introduction

Excessive intake of energy, salt, saturated fat and sugar leads to an increased risk of chronic diseases, such as cardiovascular diseases, cancer and diabetes [1,2,3,4]. In 2004, the World Health Organisation challenged the private sector to improve the food supply and reduce levels of energy density, trans fat, saturated fat, sodium and added sugar in their products [5]. Since then, reformulation has been high on the agenda of public health communities, governments and food companies. Reformulation has been defined as the “reduction of the amount of negative nutrients in (processed) foods such as energy, salt, saturated (and trans) fat and sugar” (modified from the National Heart Foundation of Australia) [6]. In The Netherlands, the reduction of energy, salt, saturated fat and sugar in processed food, either through legislation or voluntarily by the food industry, has been discussed for the last ten years, with very little progress [7,8,9]. In 2014, the Dutch government agreed with the food sector to decrease the levels of energy, salt, saturated fat and sugar in food products (National Agreement to Improve Product Composition: salt, saturated fat, sugar (calories)) [10]. The food sector is taking action to reformulate its products voluntary. Reformulation will only be successful if reformulated foods not only fit into a healthy diet, but are also of high quality and good texture, safe, good-tasting and low-priced, as described by Jiménez-Colmenero [11] for low-fat meat products and by Vandamme and Strubbe [12] and Laguna et al. [13] for low-fat sweet bakery products.

Reformulation has been the subject of many different studies in the areas of nutrition and health, legislation, food technology or consumer science. There are a number of studies on food reformulation, dealing with either technological aspects of sodium, sugar or saturated fat reduction [11,12,14], consumer aspects, such as food choice and human behaviour [15,16], or legislative aspects, such as claims about the nutritional quality of foods [17,18].

There are very few studies that take an inter-disciplinary approach to analysing the barriers and enablers of product reformulation. However, food technology and consumer perspectives have been integrated to develop a concept of consumer-driven food product development [19]. Grasso et al. [18] discussed not only technological aspects, but also legislation and consumer perceptions of reformulation in meat products; and Buttriss [20] combined public health challenges of diets and technological aspects of reformulation. These studies illustrate the value of a multidisciplinary approach to analysing the technical, legal, and social challenges to product reformulation.

**Figure 1:**
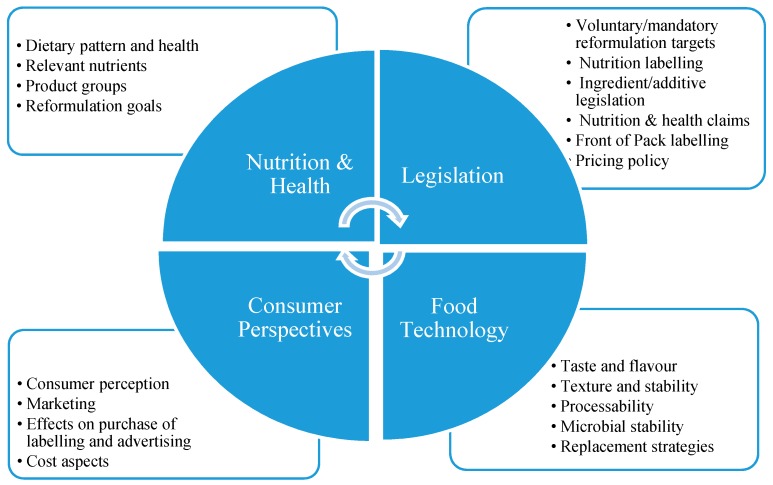
The four disciplines of a reformulation process and main topics per discipline. Disciplines: Nutrition & Health, Food Technology, Legislation and Consumer Perspectives [21].

In agreement with this, we have proposed the ‘Framework for Reformulation’, as an integrated approach of four disciplines: Nutrition & Health, Food Technology, Legislation and Consumer Perspectives (Van de Velde, Van Gunst and Roodenburg) [21]. In Figure 1, the four disciplines of a reformulation process (the Framework) are shown. Drawing on the existing literature, we have proposed the ‘Framework for Reformulation’ as an approach that integrates factors that food companies must consider in undertaking product reformulation efforts from different domains. This paper seeks to empirically test whether the Framework accurately reflects the issues taken into consideration by food companies when undertaking product reformulation.

Therefore, the aim of the present qualitative study was to determine whether this ‘Framework for Reformulation’ accurately reflects the challenges faced by food companies in undertaking product reformulation.

## 2. Methods 

### 2.1. Design

For this study, a qualitative method was chosen because the research area was relatively new, especially with respect to the integration of the four disciplines [22,23].

#### 2.1.1. Study Group and Procedure

The HAS University of Applied Sciences has a well-developed network of food companies, mostly SMEs (small and medium-sized enterprises), contained in a database. From this database, 33 companies were selected, based on the known interest and activities of companies in reformulation of salt, saturated fat and/or sugar in the bakery, meat or convenience food sector. Per sector, a list of companies and contact persons was compiled. The bakery sector was sub-divided into bread and bakery products because reformulation targets were different. First, the selected companies were contacted by phone to identify company experts in reformulation; the expertise of the experts was discussed in the interviews. These experts were informed about the study, after which a recruitment letter was sent by email. If necessary, additional information was provided. Of the 33 companies that were approached, 17 agreed to participate. Identified experts had different job titles, depending on the company type and size. Most of the companies that did not participate were too busy during that period of the year (November–December). Experts gave their written permission to be interviewed. Shared information could not be traceable to them and/or their company and it was agreed that experts spoke as individual, and not as a representative of the company. Table 1 contains details about the participating food companies and the interviewed experts. In total, nine companies in the bakery sector, five meat producers and three companies in the convenience sector agreed to participate. Because of the diverse nature of the bakery sector (bread, sweet bakery products), more companies in this sector were interviewed.

#### 2.1.2. Choice of Different Food Sectors

We selected the sectors (bakery, meat and convenience) based on their active involvement in the reformulation of food products in The Netherlands. Furthermore, their products contribute substantially to the salt, saturated fat and sugar intake of the Dutch population. Bread is responsible for 26–28% of population salt intake, meat (products) for 10–15% and bakery products for 4–5% [24,25]. Meat is responsible for 18% of saturated fat intake, sweet and bakery products for 13% [24,25] and sweets and bakery products are responsible for 17% of sugar intake [26].

#### 2.1.3. Interviews

For this study, a list of topics was developed based on preliminary examination of available literature and the ‘Framework for Reformulation’, as described by Van de Velde, Van Gunst and Roodenburg [21]. Interviews were conducted using a semi-structured topic list between November 2014 and January 2015. Table 2 contains the key themes and topics. During a first pilot interview (excluded from subsequent analyses), both the list of topics and the interview technique were tested. The key theme ‘Introduction’ was added to the list of topics. It became clear that it was important to check during the interviews whether the information was understood properly. All interviews were recorded and transcribed.

### 2.2. Data Analyses

The open coding approach was used to analyse data. Based on preliminary examination of all data, a coded list was developed to analyse the transcribed interviews. Three different researchers independently coded interviews with the aid of this list, resulting after discussion, in a clear and coherent content analysis system for all interviews [22,23]. Data were analysed in different phases: open coding (fragmentation), axial coding (coding interviews with a developed data system (key themes and topics)). With this system it was possible to select data in general or by sector and it was possible to look for statements and to make connections (selective coding) [27]. Afterwards the analyses were integrated to answer the research question.

#### Data Saturation

During the process of data collection, the subject of data saturation was considered. All themes and the list of topics (Table 2) were covered and the information from the interviews was clear for the bakery and convenience sectors. During the coding process of the last interviews, no new codes were added. In the meat sector was evident that after analysing four interviews that the information for this sector was not clear and complete (unclear information: clean label, role of government and retailer, claims and logos). Therefore, another interview was organised to obtain the missing information. Afterwards, the information was clear and data saturation was achieved in all sectors.

## 3. Results

First Section 3.1 summarizes the key incentives for and key barriers to reformulation, as derived from the Framework for Reformulation, while the following paragraphs will go into these findings in more depth.

### 3.1. Incentives and Barriers for Reformulation

The most important incentives that were mentioned were the consumer or retailer demands. Other incentives that were mentioned for the meat sector: voluntary agreements within the sector and a proactive attitude towards reformulation, because companies want to stay ahead. In the bakery sector were mentioned: the desire to join international trends, legislation and market leadership and—to a lesser extent—the desire to increase quality. The convenience sector also mentioned the desire to join international trends (healthy/sustainable diets) as an incentive.

The barriers mentioned by most companies included a fear of reduced taste, lower product quality due to replacement of functional ingredients and increasing costs due to alternative functional ingredients or changes in processing. An additional barrier, as previously mentioned, was the ‘absence of a level playing field’, because not all companies in the sector reformulate.

### 3.2. Food Technology

Table 3 contains an overview of the food technological aspects that were mentioned in the interviews. Target nutrients for reformulation, their function, technological solutions and challenges, as mentioned in the interviews, are presented per sector. This Table also provides information about the frequency target nutrients for reformulation were discussed in the interviews per sector.

The three sectors differed in their main target nutrients for reformulation and challenges. In the sweet bakery sector, these are sugar and saturated fat. In the bread, meat and convenience sectors, it is salt. Sugar and saturated fat were difficult to substitute in the sweet bakery sector as they are bulk ingredients. In the meat sector, it was difficult to substitute salt and to keep the shelf life and sensory quality acceptable. The convenience sector had difficulties with the replacement of sugar with Stevia, which is unstable when heated. A common theme was ‘clean label’ or E-number reduction. For companies, the declaration of E-numbers on food labels is mandatory and can be done by using their common (chemical) name or their E-number [28]. Cleaner labelling was described as “being produced as free of chemicals, having easy-to-understand ingredients lists and being produced by use of traditional techniques with limited processing” [29]. So ‘cleaner labelling’, a trend in the food industry, eliminates chemical sounding ingredients or ingredients recognised as artificial in food products [30,31].

*“What our company does well is to reduce the amount of E-numbers. In ‘Filet Americain’ we reduced them from 21 to 7, which is positive”*.

*(Company in the meat sector)*

*“We are reducing E-numbers in our products. Customers frequently ask if there are E-numbers in our products. We use as few E-numbers as possible. We already have a product concept for E-number-free bread and sausage rolls. Adapting a recipe is primarily adjusting the raw materials, as these contain the E-numbers”*.

*(Company in the bakery sector)*

Companies used as few E-numbers as possible, due to demand from both retailer and consumers. Retailers used their own colour-coded lists of E-numbers to make a distinction between those that should not be used at all, those that can be used only when there is an added value and those that can always be used. E-numbers have a bad reputation with consumers, although according to interviewees, technologically it might be better to use these E-numbers, which are approved for use in foods.

*“For a long time our statement was: E-numbers are approved additives and required to make a good and sustainable product. But this year we implemented an E-number policy: nowadays, our company has a classification system with red, orange and green. Red E-numbers in food products must be replaced. The company leaves the orange E-numbers in the products for as long as the private label customer does not object. When the customer objects, we take these E-numbers out. In case of new products, with clean label as unique selling point, we do not use orange E-numbers, but only the green ones”.*

*(Company in the convenience sector)*

At least half of the interviewed companies from all sectors had some ’clean label’ products. Sometimes, their focus was on ’natural’ E-numbers, which were deemed acceptable. Although in legislation there is no difference between ‘natural’ and ‘artificial’ E-numbers [32], consumers think that ‘natural is safe’. Consumers use ‘natural’ as a simple feature that labelled on food products show that these products contain superior attributes and are perceived to be less harmful and healthier than conventional [33]. Although the price for natural ingredients was generally higher, companies reported that these products were selling well because of the positive narrative attached to them. However, in the meat sector higher prices were not acceptable.

*“Reformulated products should be sold. They are somewhat more expensive, but there is a positive story attached to them and that is selling well”.*

*(Company in the bakery sector)*

In summary, the different sectors differed in their focus with respect to reformulation. Participants mentioned various technological challenges, which varied between the sectors. A frequently mentioned common theme was ’clean label’. Based on consumer and retail demands, most companies were removing E-numbers from their products.

### 3.3. Nutrition & Health

#### 3.3.1. Health Impact through a Healthy Product Range

All companies mentioned the importance of health aspects, but most did not investigate the health impact of their products because of limited time or lack of budget. In the *meat sector*, the importance of health aspects is increasing. However, it was difficult for some products to be positioned as ‘healthy’, such as traditional meat products. Consumers wanted to have meat products as ‘pure’ as possible with no additions. The meat companies were apart from the current reformulation goals, not actively involved in making the food supply healthier, with two exceptions. One company wanted actively to improve the food supply for children by reducing salt in products for children. Another company developed a product with less meat and added vegetables to increase vegetable consumption. In *the bakery sector*, interviewees reported differences between bread and sweet bakery products. Bread is a basic food and a source of protein and fibre, salt has been reduced. Sweet bakery products, on the other hand, are luxury products, which will be eaten occasionally. For those products, taste is more important than health-promoting positioning. The *convenience sector* reported the development of a range of healthy products. Companies created variation in diets by using cycles of menus and offering consumer choice through new technologies, for example, by adding raw vegetables to pre-steamed meals, in which vitamins and minerals are better preserved.

#### 3.3.2. Communication about Nutrition and Health

Some studies suggested that reformulation efforts need to be supported by consumer education in order to be effective [20]. This was also mentioned in the interviews. All interviewed companies agreed that communication about nutrition and health to consumers needed to improve, but in their solutions, they differed on who should be involved: The Netherlands Nutrition Centre (government), the food industry or both. Participants said that consumers might be overloaded with information about the nutritional quality of food products and that a label or a logo on products could assist consumers in interpreting this information. Companies in all sectors mentioned that education about nutrition and health should be given at all primary and secondary schools. Many participants did not communicate directly to consumers about the health aspects of their products; they delivered their food products to a retailer and were dependent on the retailers’ communication. One company communicated to the retailer through labelling on package and to customers in the food service through journals and social media. A minority of *meat companies* mentioned that communication about the health aspects of their products could have the opposite effect to consumers (it could discourage them from eating reformulated products) and that a slow, stepwise decrease of salt was the best strategy. In the literature different strategies were described for salt reduction, one of these was a step-by-step reduction. This has been applied to reduce sodium in bread [21,34]. Other strategies mentioned in the literature were: use of sodium replacers, use salt enhancers and salt boosters (such as yeast, flavours) and the inhomogeneous distribution of salt in a food product, stimulating of taste receptors [21].

*“We do not communicate to consumers that we reformulate our products. …Once salt is reduced in the product, people will notice. We just want to decrease salt slowly, so that the consumers notice as little as possible”.*

*(Company in the meat sector)*

In summary, based on their different product portfolios, companies differed in their focus on health. For bread and meat producing companies, health was a logical focus. However, sweet bakery products were considered as treats and meat products as traditional—both diverging from a health positioning. It was a generally held opinion that communication to consumers about nutrition and health should be improved, starting at schools. Most companies maintained a conservative approach to communicating their improvements on reformulation for fear of consumers rejecting reformulated products.

### 3.4. Legislation

Some of the companies in the convenience sector were against strong interference by the government. However, all meat companies and half of the bakery companies mentioned that they would like the government to strengthen reformulation efforts. In their opinion, the government or industry associations should push for legislation when existing covenants are not respected, requiring all companies in the sector to participate, thus creating a level playing field.

*“The government should enforce companies to reformulate their products. We need an independent stakeholder. There are so many different interests.”.*

*(Company in the bakery sector)*

*“The role of government is very important in reformulation. If the government does nothing and some companies want to tackle the issue and others not, the companies that want to reformulate are at a disadvantage. So, there should be regulation. Everyone should be able to play on the same playing field without problems.”.*

*(Company in the convenience sector)*

Most companies were familiar with the ‘National Agreement to Improve Product Composition’ [10]. Meat companies used it as a basis for product reformulation. Companies in the bakery and convenience sector mentioned that they already met its requirements. Pressure from the government was felt in the bakery sector, but not in the meat and the convenience sector. The bakery sector had taken up the responsibility to reduce salt in bread, after which the government aligned the relevant legislation with existing industry efforts. This was considered a good example of salt reduction initiated by the sector and by retail organisations.

#### 3.4.1. Legislative Restrictions

Most companies encountered no restrictions from Dutch or foreign legislation on their reformulation processes. However, some restrictions were mentioned: protected product names, organic products, prohibition of Stevia (Steviol glycosides or E 960) in bakery products and foreign legislation, which differed in labelling and requirements for the use of flavourings.

#### 3.4.2. Food Labelling, Claims and Health Logos

Food companies may reformulate products in order to enable them to put a logo on products or supportive product labelling may form an important part of the reformulation programs. The companies in the meat and bakery industries mentioned no issues about the new labelling legislation (Regulation EC 1169/2011) [28]. This EU regulation describes what information is obligatory on the food labels of pre-packaged foods, such as the ingredient declaration and nutrition table (per 100 g). Companies in the convenience sector reported some difficulties with the implementation of this legislation due to the big variety of products in their portfolio. Companies reported using ‘health claims’ sparingly. Some companies used ‘nutrition claims’ (Regulation EC 1924/2006, health and nutrition claims) [35]. This EU regulation describes the conditions for using health claims (such as ‘lowers your cholesterol’) and nutrition claims (such as ‘low fat’ or ’reduced salt’) on the package of food products. Table 4 summarizes the reported use of nutrition claims and health logos and the main reasons why companies chose not to use these claims or logos. Health logos are also voluntary, “the Choices logo” in The Netherlands (disappearing) or in the UK “traffic light” system for Reference intakes. Table 4 shows that nutrition claims were used across all sectors, while the type of claim differed per sector. Few companies in the meat sector and none of the companies in the bakery and convenience sectors carried a health logo to help consumers make healthier choices. Most interviewed meat and bakery producers did not use this logo because they only produced private label products. In the meat sector this logo was not seen as useful. The meat sector reported focusing on obtaining the ‘animal welfare-logo’.

For sweet bakery products, it was very difficult to comply with the criteria of the health logo. Several sweet bakery companies reported reformulating their products to obtain a health logo in future. One company mentioned that it was not justified to use health logos on indulgent products. In the *convenience sector* companies reported that they had their own ‘health’ criteria or that they had too many different products to use nutrition and health claims. (Table 4).

In summary, most companies mentioned that they see a key role for the government. Agreed common goals would stimulate a level playing field. Most companies agreed that the government should be encouraging reformulation to a greater extent. Some restrictions to reformulate caused by legislation were mentioned. The new labelling legislation required a major effort for some, but was no longer an issue. Nutrition claims were possible, but were used only occasionally. Health logos were hardly used by any of the participating companies.

### 3.5. Consumer Perspectives

#### 3.5.1. Consumer Trends

Companies thought that the knowledge of consumers about healthy foods was limited and that consumers were poorly informed by the media and the Internet. They thought that consumers were confused and that, suspicion and emotional arguments determined their choices. However, according to the interviewed companies, only a small percentage of consumers read the package labels.

The market for meat products was considered as traditional: 60% of consumers always bought the same meat products. Animal welfare and E-numbers (Clean label) were reported as important issues. Most meat companies mentioned that consumers did not really influence reformulation, because only a small minority pushed for clean labels. According to the companies in the bakery sector, trends such as super foods, spelt, portion size and clean labels, were translated into retailer demands. They stated that consumers increasingly asked for clean labels. Therefore, some companies focussed on reducing E-numbers. However, taste aspects remained the most important and companies were concerned whether a healthier cookie would still taste good. Clean labels also became increasingly important within the convenience sector. Additional trends mentioned were vegetarian products and the use of insects.

#### 3.5.2. Pricing and Branding

Lack of branding was perceived as slowing down reformulation in the Dutch meat sector. Through brands, companies could distinguish themselves with reformulation, as was the case in France. Being a leading company in reformulation and pricing were important issues. In the bakery sector, reformulated and clean label products were more expensive and, therefore, not being sold. Because some competitors, which did not invest in reformulation, had no disadvantage and benefitted by offering their products at a lower price. Therefore, some companies wanted the government to legislate and create a level playing field.

#### 3.5.3. The Role of the Retailer in Product Reformulation

According to meat producing companies, retailers had significant power to influence the food supply. Through tenders, supermarkets determined which private label products to introduce. The companies stated that they did not know what the retailer would accept in terms of price and sensory aspects. Clean labels were important to retailers as they had requirements on E-numbers and did not want to be associated with food scandals. In the bakery sector, the role of the retailer varied: retailers determined what was on the shelves. Companies developed E-number free products, but the higher price was a problem. Retailers were the driving force behind reformulation in the bakery sector. For E-numbers they used the colour coded lists, as previously mentioned. Consumer organisations were also pushing for such lists. Companies in the convenience sector felt a similar pressure from retailers about clean labels. So, in all sectors the pressure for reformulation from retailers was big because they determined which products will be introduced in supermarkets.

#### 3.5.4. Sensory Requirements

Consumer trends in taste were tracked by companies or by external agencies. Examples included testing sodium-reduced products, opinions about claims and performing concept studies. According to retailers, reformulated products in the bakery and meat sectors should score at least equivalent to regular products. According to interviewees, taste was always the primary consideration for consumers.

*“Sensory properties are interrelated. You can manipulate a lot with aroma, colour and taste. That is what we do. Number 1 is that it is a good and tasty product”.*

*(Company in the bakery sector)*

*“A snack should taste good. That is the customer’s main requirement. Sensory quality is the most important. Reformulated products should not deviate from regular products regarding sensory aspects”.*

*(Company in the meat sector)*

One company in the bakery sector had already marketed a successful reformulated product. Natural products were selling very well. Interestingly, sweet bakery products used to contain more fat, but consumers had accepted a gradually-lowered fat content. In the convenience sector, reformulated products were required to look the same as regular products, and should taste good—even if a similar taste is not possible.

In summary, clean labelling was the consumer trend mentioned most frequently as influencing reformulation. This determined the key role in retailer demands for product development. Requirements such as similar taste and costs have hampered reformulation, preventing companies from obtaining a return on their reformulation investment. This was further hampered by lack of branding when companies were producing for a private label.

## 4. Discussion & Conclusions

The aim of this study was to determine whether this ‘Framework for Reformulation’ accurately reflects the challenges faced by food companies in undertaking product reformulation.

The interviews illustrated that the four disciplines of the reformulation process: Food Technology, Nutrition & Health, Legislation and Consumer Perspectives play an important role in the reformulation of food products.

Concerning the discipline Food Technology, interviewed food companies in all sectors mentioned various solutions to replace the functionality of salt, saturated fat and sugar to obtain reformulated products of comparable quality. Salt reduction was easiest. This is confirmed by research on various European salt reduction initiatives [36]. Reduction of saturated fat and sugar was reported to be more difficult, especially in the sweet bakery sector, because both are bulk ingredients. Reduction of these ingredients in sweet bakery products alters the structure, flavour and mouth feel [12].

Nutrition & Health aspects are important drivers of a reformulation process, since excessive intakes of energy, salt, saturated fat and sugar lead to an increased risk of chronic diseases, such as cardiovascular diseases, cancer and diabetes [1,2]. In this study, food companies in all sectors mentioned opportunities for reducing the salt, saturated fat and sugar content of their products, but they also focussed on E-numbers. The reduction of E-numbers (clean labels), as well as sensory and price aspects, appeared to be more important than the health impact of products. However, E-numbers do have a function in foods and many are available for product optimisation to reduce the salt, (saturated) fat and sugar content [20,37,38]. Therefore, it is possible that the use of E-numbers can make the food supply healthier (i.e., substitution of sweeteners for sugar) and clean labels can potentially lead to less healthy products and shorter shelf life [38]. Thus efforts to reduce the use of E-numbers may in fact be undermining the healthiness of products.

The domain of Legislation has two important aspects: (voluntary) reformulation of food products and the possibility of using nutrition and health claims or logos to distinguish between regular and healthier products. At first, in 2014, the Dutch Government came to an agreement with the food sector to lower voluntarily the levels of salt, sugar, saturated fat and energy density in food products [10]. In the interviews, we have seen that this agreement had a limited effect on the reformulation processes within companies. This was confirmed by studies in The Netherlands [7,8,9]. Most companies also mentioned that the government should be further encouraging reformulation for example through legislative measures, to create a level playing field. Secondly, this study discovered that most smaller companies did not frequently use nutrition claims and health logos. As a result, they may be missing a good opportunity to communicate information about their reformulated products to consumers. Larger companies would possibly use more logos and nutrition claims, but this was not measured in this study. European consumer studies conclude that consumers look mostly at nutrition labels and Reference Intakes and find those important. However, this did not stimulate them to make healthier choices [39,40]. Nevertheless, in another study, health logos seemed to enhance healthier product choices, even under time pressure [41]. Most of these studies used a questionnaire and did not look at actual consumer behaviour. Whether nutritional information stimulates consumers to buy healthier products was examined in only a few studies, which observed consumers in the supermarket [42] or recruited consumers shortly after they had bought their foods [43]. These studies show that few consumers pay attention to the nutritional information and are persuaded by that information to buy healthier food products.

Despite this Consumer Perspectives are important, according to the interviewed food companies. Taste (sensory aspects) and price are most important, but health is also a salient concern to consumers. Literature confirmed that sensory properties such as taste, smell, texture and flavour—as well as visual and auditory aspects—have the most powerful influence on consumers [15,44]. In addition, ‘health’, ‘convenience’ and ‘price’ are also important [15]. In the present study, food companies also mentioned that consumers want to have fewer E-numbers in food products as has been found in other studies. Even though E-numbers are approved for use in foods, various studies have reported the negative perception of E-numbers by consumers [45,46,47], possibly caused by a lack of trust in the food sector. Consumers regularly consider E-numbers as unnatural, artificial and detrimental to their health [46,47]. Therefore, they ask for ‘natural’ and unprocessed products without E-numbers [45].

From the interviews, it appeared that retailers, price and the push to reduce E-numbers (clean labels) were also important and these aspects must be included in the Reformulation Framework (Figure 1). Retailers are key players and play a central role in improving the food supply in two ways: by determining what is offered to the consumers in supermarkets and by controlling the private label production. This is illustrated by The Netherlands Environmental Assessment Agency. In The Netherlands, only five purchasing organisations from 25 supermarket chains serve 17 million consumers [48], which demonstrates the control retailers have on market availability. Finally, reformulation is not only about lowering salt, saturated fat and sugar levels, but also about the reduction of E-numbers. From our study, it appears that nearly all food companies are discussing the reduction of E-numbers in foods and that quite a few of these companies already have marketed products with fewer E-numbers. It is important to study the effect of reformulation of E-numbers. Are food companies reducing E-numbers, as well as salt, sugar and saturated fat, or does the reduction of E-numbers lead to a higher amount of salt, saturated fat and sugar in foods?

### 4.1. Strengths and Limitations

This qualitative study is one of the first to determine whether this ‘Framework for Reformulation’ accurately reflects the challenges faced by food companies in undertaking product reformulation. During our research, attempts have been made to minimise subjectivity and optimise importance by selecting companies from sectors that are relevant to reformulation in The Netherlands: the bakery, meat and convenience sectors.

In this study, both the interview technique and the list of topics were tested before the start of the interviews (and thus, excluded from further analyses). Coherence in the analysis was assured through independent coding of the interviews by three researchers.

During this study, data saturation has been considered. At the end of coding process, all themes and topics (Table 2) were covered and the information from the interviews was clear for all sectors. During the coding process of the last interviews, no new codes were introduced. This study concerned semi-structured interviews with representatives from 17 Dutch food companies in the meat, bakery and convenience sectors. These companies were known to be active in reformulation and are only representative for their sector. It must be noted, however, that the companies do not necessarily reflect the entire Dutch food sector. This study provides only an indication of the most important aspects of the reformulation process, as perceived by these food companies. Results in other sectors or in bigger companies could be different, for example in using nutrition claims or health logos. Further on the processes of reformulation in other European countries or the USA could be different.

For this study, a list of topics was developed based on preliminary examination of available literature and the ‘Framework for Reformulation’, as described by Van de Velde and Van Gunst and Roodenburg (2016) [21]. All four disciplines seem to be relevant for food companies, but during the interviews a fifth ‘perspective’ was mentioned in nearly all interviews: the retail perspective. In this study, the definition of reformulation was limited to salt, saturated fat and sugar. However, some food companies employed a broader definition of reformulation: including E-numbers as well as some other target nutrients (see column 1 of Table 3).

### 4.2. Recommendations

Since all four domains play an important role in shaping companies’ reformulation-efforts, policy makers should give consideration to the factors considered in this Framework for Reformulation when developing product reformulation programs. Additional research is needed to determine whether and how this ‘Framework for Reformulation’ can be used as a basis for actual reformulation processes in food companies. An intervention study within food companies would be necessary for this type of research. Another potential research topic is whether the focus on E-number reduction in food companies distracts the food industry from reducing the salt, saturated fat or sugar content of food products. For this research question, a quantitative study of reformulated foods would be necessary.

It can be concluded that this study suggests that all four disciplines of the Framework for Reformulation: Food Technology, Nutrition & Health, Legislation and the Consumer Perspectives play an important role in the reformulation of food products by food companies. It can also be concluded that reformulation is not only about lowering the salt, saturated fat and sugar levels because of their proven association with chronic diseases. According to food companies, it is perhaps even more related to reducing E-numbers in products, due to the alleged distrust of consumers in these substances despite their approval for use in foods. Retailer as well as consumer perspectives shaped reformulation efforts, meaning that the influence of retailers should be included as a dimension of the framework. The research also seems to suggest that there is demand among food companies for greater government support for product reformulation, for example in the form of regulation that requires all companies to reformulate their products.

## Figures and Tables

**Table 1:** Study group food companies (*n* = 17): sectors, number and size companies, main products and employment of interviewed experts.

Sector	Number of Companies	Number of SME Companies (<50 Million in Sales Revenue or <250 Employees)	Number of Bigger Companies (>50 Million in Sales Revenue or >250 Employees)	Main Products	Job Title of Interviewed Experts
Bakery	9	6	3	5 Biscuit/Banquet/Cake/Pastry 2 Bread and sweet bakery products 1 Ingredient supplier for Bakery products 1 Food bars	4 Product developers 3 Managers/directors 1 Product- and process developer 1 Quality and product manager
Meat	5	3	2	2 Processed meat 1 Semi-processed meat products 1 Snack products 1 Ingredient supplier for the meat sector	3 Product developers 1 Director 1 Quality manager
Convenience	3	2	1	2 Meals (soups and sauces included) 1 Products for catering (institutions/private)	1 Director 1 Product developer/dietician 1 Quality manager

**Table 2:** List of key themes and topics on reformulation (salt, saturated fat, sugar) for interviews in food companies.

Key Themes Reformulation	Topics Reformulation
Introduction	Function expert Size, activities, mission company Main food products
General aspects	Experiences company Main opportunities Main barriers Pressure from other companies, government or FNLI (Federation of Dutch Grocery and Food Industry) to reformulate
Food Technology	Main technological nutrients and principles Challenges in reformulation (new technologies) Reformulated products on the market Experiences Problems during reformulation Quality and food safety reformulated products Use of additives Sensory conditions (taste, structure, texture) and conditions for shelf life
Nutrition & Health	Making food supply healthier Importance of health aspects Impact of food products on consumer health Partnerships with food companies, government or research institutes Reformulation in other nutrients
Legislation	Legal restrictions Labelling requirements Use of nutrition & health claims and advantages or disadvantages Use of health logos Taking into account the Agreement on Improvement of Product Composition Governmental measures to enforce companies to reformulate
Consumer Perspectives	Role of consumers in company decision to reformulate Responding to (which) consumer trends Communication to consumers about reformulated products Reaction of food companies to consumer demand for clean label products Consumer reaction to reformulated products Consumer buying behaviour according reformulated products Price aspects
Other relevant aspects	Not mentioned aspects/relevant knowledge of companies

**Table 3:** Overview of food technological aspects that were mentioned in the interviews: target nutrients for reformulation, their function, technological solutions and challenges. In total, 17 companies from bakery (*n* = 9), meat (*n* = 5) and convenience (*n* = 3) sectors.

Target Nutrient(s) Reformulation	Function Target Nutrient	Reformulation Solutions	Technological Challenges
Bakery sector (*n* = 9)			
Sugar reduction (sweet bakery products (*n* = 4) *	Preservative, taste, bulk, structure, brown colouring	Addition of sweeteners and alternative bulk ingredients, such as fat	Difficult to replace as bulk ingredient; substitution with sweeteners decreases shelf life (risk of spoilage) & changes of the dough and product structure
(Saturated) fat reduction (sweet bakery products (*n* = 4)	Bulk, structure, taste	Reduction of total fat; substitution with unsaturated fat from rapeseed oil or sunflower oil; addition of additives	Difficult to replace as bulk ingredient, difficulties to structure different fat phases, leading to impaired product quality; higher costs of unsaturated fat ingredients
Salt reduction (bread (*n* = 3)	Taste, dough structure	Substitution with potassium chloride (E508), monosodium glutamate, MSG (E621); addition of bread improver ^b^ (no E-number)	Successfully lowered
E number reduction (*n* = 4)	Various	Innovative technique to replace oxygen in packages; Replacement by natural alternatives.	Concern about microbiological safety; natural colourings are unstable; emulsifiers are difficult to replace; costs are higher.
Addition of whole grain, oat, spelt, fibres, nuts, protein, vitamins, antioxidants and calcium (*n* = 6)	Health benefits	None mentioned	None mentioned
Meat sector (*n* = 5)			
Salt reduction (*n* = 4) *	Preservative, taste	Substitution of salt with potassium lactate (E326), natural minerals; gasification; addition of antioxidants (vitamin C or E: E300, E308); heat treatment; high pressure processing (HPP ^a^)	Difficult to substitute salt and maintain good shelf-life & sensory quality at acceptable costs and not use E numbers in ’clean label’ products.
(Saturated) fat reduction (*n* = 5)	Structure, taste	Substitution of fat with water; extrusion; addition of fibres and starches. Snacks: use of ‘air fryer’ (out of home market)	Efficiency and costs without use of E-numbers in ’clean label’ products.
Sugar reduction (*n* = 1)	Brown colouring, binding with proteins	None mentioned	None mentioned
E number reduction (*n* = 1)	Various	None mentioned	Costs are often higher, higher price is a problem. Efficiency and costs are challenges
Protein enrichment (*n* = 1)	Structure	Addition of protein to bind water and fat for firmer easier to slice meat	None mentioned
Convenience sector(*n* = 3)			
Salt reduction (*n* = 2) *	Taste	Substitution with taste intensifiers, such as herbs, MSG (E621) or potassium chloride (E508)	Mostly easy, sometimes a challenge (good taste)
Sugar reduction (*n* = 2)	Taste	Sugar reduction (30%) in 3 years. Substitution with sweeteners (Stevia, Steviol glycosides, E960)	Stevia is not heat stable and has a bitter taste; alternative sugar substitutes and techniques are needed
E number reduction (*n* = 2)	Various	Substitution with ’natural’ alternatives	Costs are often higher
Enrichment with vitamins and minerals (*n* = 2)	Health benefits	Use of raw materials, mild processing (better preservation vitamins)	Concern about the microbiological safety/shelf life of ultra-fresh meals

^a^ HPP: cold pasteurisation with high pressure to inactivate microorganisms; ^b^ Bread improver disappeared biochemically during bread preparation, so it does not have to be mentioned on the label; * (*n* = *x*) number of companies per sector, that mentioned target nutrients in the interviews.

**Table 4:** Used nutrition claims and *health logos* and reasons not to use these, as mentioned in the interviews with 17 companies from the bakery (*n* = 9), meat (*n* = 5) and convenience (*n* = 3) sectors.

	Meat (*n* = 5)	Bakery (Only Sweet Bakery) (*n* = 9) *	Convenience (*n* = 3)
Nutrition claims/Health logos used **	Low sodium (*n* = 1) Health logo (*n* = 2) Focus on animal welfare-logo (*n* = 3)	Source of fibre (*n* = 3) High-fibre (*n* = 1) Gluten-free (*n* = 1) Less sugar (*n* = 2) Calcium-fortified (*n* = 1) Light (*n* = 1) Products for target groups: extra calcium, probiotics (*n* = 1)	High fibre (*n* = 1) Source of fibre (*n* = 1) Low sodium (only for target group) (*n* = 1)
Reason not to use nutrition claim or health logos **	Negative consumer perception, suspect tinkering with products (*n* = 1) Traditional product (*n* = 1) Private label production, retail decides (*n* = 1)	Indulgence products with limited health benefits (*n* = 1) Private label production, retail decides (*n* = 1) Comparative claim ‘less sugar’ was perceived as unclear regarding the ‘reference’ product (*n* = 1) Difficult to comply with criteria health logo *(n* = *1)*	Limited understanding by consumers (*n* = 1) Limited space for claims on packaging (*n* = 1) Many products, too much work (*n* = 1)

* No claims and logos were used on bread; ** number of companies (*n* = *x*) per sector that mentioned this aspect in their interviews.
